# Histopathological Verification of Abnormal Cytology Results Suggesting High-Grade Intraepithelial Lesions in Women over 50 Years of Age—Evaluation of the Clinical Utility of Conventional Gynecological Cytology

**DOI:** 10.3390/jcm14238305

**Published:** 2025-11-22

**Authors:** Wiktoria Utkowska, Brygida Tucka, Jakub Szyszkowski, Krzysztof Krasuski, Artur Ludwin, Barbara Suchońska

**Affiliations:** 1 1 st Department of Obstetrics and Gynecology, Medical University of Warsaw, 02-015 Warsaw, Poland; s082760@student.wum.edu.pl (B.T.); s082747@student.wum.edu.pl (J.S.); artur.ludwin@wum.edu.pl (A.L.); barbara.suchonska@wum.edu.pl (B.S.); 2 Department of Medical Informatics and Telemedicine, Medical University of Warsaw, Litewska 14/16, 00-581 Warsaw, Poland; krzysztof.krasuski@wum.edu.pl; 3 Faculty of Mathematics and Information Science, Warsaw University of Technology, Koszykowa 75, 00-662 Warsaw, Poland

**Keywords:** cervical cytology, HSIL, histopathology, postmenopausal women, diagnostic accuracy

## Abstract

**Objectives**: Cervical cancer remains a major health concern worldwide. In women aged ≥ 50, diagnostic accuracy may be compromised due to menopausal changes such as atrophy and squamocolumnar junction displacement. Cytology remains the primary screening tool in many regions, including Poland, although its sensitivity and specificity are limited. This study assessed the concordance between cytological diagnoses of high-grade squamous intraepithelial lesions (HSILs); atypical squamous cells, which cannot exclude HSIL (ASC-H); atypical glandular cells (AGCs); and histopathological verification in women aged ≥ 50 years, highlighting the limitations of current diagnostic pathways. **Methods**: A retrospective analysis was conducted on women aged ≥ 50 years referred between 2018–2024 with abnormal cytology. All patients underwent colposcopic assessment followed by histopathological verification supported by p16 immunostaining. Cytological and histopathological results were compared. Associations between clinical variables and diagnostic concordance were tested using the chi-square test (α = 0.05). **Results**: Among 79 patients, histopathology confirmed high-grade squamous intraepithelial lesions with cervical intraepithelial neoplasia grade 2 or higher (HSIL/CIN2+) in 38%. Low-grade squamous intraepithelial lesions with cervical intraepithelial neoplasia grade 1 (LSIL/CIN1) were found in 11%, and vaginal intraepithelial neoplasia grade 1 (VAIN1) in 4%, while 47% demonstrated inflammatory changes or no abnormalities. HSIL cytology showed the highest concordance, whereas AGC was more frequently associated with benign findings. No statistically significant association was detected between cytology accuracy and clinical characteristics (*p* > 0.05), highlighting the need for further studies in larger cohorts. **Conclusions**: In women aged ≥ 50, abnormal cytology frequently overestimated the severity of cervical pathology. Reliance on cytology alone may lead to overtreatment or misclassification, particularly in the presence of atrophic or inflammatory changes. Complementary use of human papillomavirus (HPV) genotyping and molecular markers alongside histopathological verification is recommended to enhance diagnostic precision in this population.

## 1. Introduction

Cervical cancer is the fourth most common cause of cancer incidence and mortality among women worldwide [1], while in Poland it ranks eighth and ninth, respectively [2]. A considerable disparity in incidence, diagnostics, and mortality is evident compared both to other European countries and on a global scale [3]. Age is one of the factors taken into consideration during the process of screening for precancerous cervical lesions within the general population. In this particular patient group, the diagnostic process may take a radical form, including the implementation of electrosurgical conization as a therapeutic procedure [4].

For many years, cervical cancer screening in Poland was based exclusively on conventional cytology performed every three years in women aged 25–59 years, within the national programme coordinated by the National Health Fund. Although this approach contributed to reducing cervical cancer incidence, its diagnostic sensitivity, particularly in postmenopausal women, remained suboptimal.

The recent guidelines published by the Polish Society of Gynecologists and Obstetricians (PTGiP) advocate a screening approach informed by high-risk human papillomavirus (HR HPV) genotyping, with a particular emphasis on identifying HPV types 16 and 18 [5]. These findings align with the recommendations put forth by international societies and national regulations [6].

Building upon these recommendations, the Polish Ministry of Health has introduced HR HPV testing and liquid-based cytology (LBC) into the national cervical cancer prevention programme as part of the 2025 update [7]. The revised protocol covers women aged 25–64 years, with HPV-negative cases recommended for retesting every five years and HPV-positive cases referred for cytological triage and, if necessary, colposcopic assessment. In Poland, a transition period will last until the end of December 2026, during which both conventional cytology and HPV typing and LBC will be acceptable [6]. This evolution of the national screening protocol aligns with international efforts to transition from cytology-based to HPV-based primary screening, aiming to improve diagnostic sensitivity and early detection of cervical precancerous lesions, particularly in women aged ≥ 50 years.

Precancerous cervical lesions are most commonly diagnosed in women aged 35 to 50 [7]. As stated in a report by the National Cancer Registry (KRN), the highest incidence of cervical cancer was recorded in the 60–64 age group [2]. This finding suggests that women aged ≥ 50 years may be more susceptible to high-grade intraepithelial lesions, which can serve as precursors to the development of invasive cervical cancer. In consideration of the natural progression of high-risk HPV infection, this demographic is predisposed to the development of intraepithelial neoplasia and, consequently, cervical cancer [1,8]. This risk is further compounded by physiological and anatomical changes that result in a displacement of the squamocolumnar junction towards the cervical canal.

The most significant etiological factor in cases of cervical cancer is persistent high-risk HPV infection [9]. A higher incidence of the infection has been observed among women who have become sexually active at an early age, have multiple sexual partners (especially in polygamous relationships), are infected with Chlamydia trachomatis, and have given birth multiple times [10,11]. It is evident that a number of other significant factors have a role to play in the development of the disease. These include low socioeconomic status and smoking [12]. Furthermore, women infected with human immunodeficiency virus (HIV) and those undergoing long-term immunosuppressive therapy are at high risk of squamous cell carcinoma [13,14].

The efficacy of secondary prevention is influenced by hypoestrogenism and menopause-related changes in the vagina and cervix, such as atrophy and upward displacement of the squamocolumnar junction to the cervical canal above the external os. Such alterations have the potential to impede the accurate procurement of endocervical cells, thereby compromising the interpretation of the resulting cytological image. This, in turn, can lead to an elevated risk of diagnostic inaccuracies and false-negative cytology outcomes [15].

Despite the fact that cytological testing has been utilized extensively for approximately eight decades and is recognized for having contributed to the preservation of millions of lives, it is important to acknowledge its limitations. The sensitivity of the test ranges from 30% to 87%, which indicates that, even in the most advanced medical facilities, up to one-third of cases of cervical cancer may remain undetected despite a normal result on the Pap test [16]. Nevertheless, in many regions of the world, including Poland, it remains the primary modality for cervical cancer screening due to its relative cost-effectiveness, expeditious nature, wide availability, and minimal invasiveness, thus satisfying the pivotal criteria for screening [5].

In countries such as Finland, the Netherlands and Australia, effective cytology-based screening programmes have almost completely eliminated cervical cancer [17,18,19], resulting in an extremely low mortality rate of 1 to 2 per 100,000 women, compared to the global average of 7 per 100,000 women [20,21]. The discrepancy in the efficacy of screening programmes across different nations can be attributed to variations in the training systems employed for cytotechnologists. This assertion pertains to both conventional cytology and liquid-based cytology. In Poland, training is relatively brief and does not encompass multi-stage certification exams, which may impact the diagnostic value of the test with regard to sensitivity and specificity.

Abnormal cytology results are not always easy to verify quickly and definitively. Positive results—high-grade squamous intraepithelial lesion (HSIL), atypical squamous cells—cannot exclude HSIL (ASC-H), or atypical glandular cells (AGC)—indicate the need for further diagnosis, such as colposcopy, a targeted cervical biopsy or cervical curettage. In postmenopausal women, electrosurgical conisation may also be necessary [22]. In some cases, the final diagnosis cannot be established during the initial evaluation; therefore, patients with HSIL cytology and a negative histopathological verification result must remain under the supervision of a specialist [23]. This situation has been taken into account in the guidelines of scientific societies dealing with cervical pathology [5].

### Aim

This study aims to evaluate the consistency between cytological and histopathological findings in the cervix of women aged ≥ 50 years who have been diagnosed with high-grade intraepithelial lesions (HSIL, ASC-H or AGC) in cytological examinations. This study aims to highlight the limitations of, and assess the effectiveness of, the diagnostic methods used to date. The study intends to contribute to a better understanding of these limitations, which may encourage gynecologists to adhere to current guidelines more conscientiously and meticulously [5].

## 2. Materials and Methods

### 2.1. Study Design and Population

A retrospective study based on the analysis of medical records collected during routine visits to the Outpatient Clinic of the 1st Department of Obstetrics and Gynecology at the Medical University of Warsaw (WUM) was conducted.

The study group comprised women aged ≥ 50 years who had abnormal cytology results classified as HSIL, ASC-H or AGC. The analysis included results obtained during advanced diagnostic tests. Data on the patients’ general and gynecological histories were also taken into account. The study included patients whose cytological smears were taken at our facility, as well as patients referred by external physicians due to abnormal cytology results. As cytological examinations were performed in different laboratories, the quality of cytological interpretations issued by individual diagnostic centres could not be assessed. Consequently, the data obtained were generalized.

Between October 2018 and March 2024, abnormal gynecological cytology results were found in 188 patients. Seventy-nine of these patients were diagnosed with HSIL, ASC-H or AGC, and they were the subjects of interest to the researchers. In accordance with the guidelines in force during the study period, these patients did not undergo mandatory HPV genotyping, but were referred for colposcopy instead. Colposcopic examination and biopsy were performed without prior administration of local estrogen therapy, in line with the recommendations of the Polish Society of Gynecologists and Obstetricians (PTGiP) applicable at that time. During the initial consultation, a colposcopy was performed and histopathological material was collected under local anesthesia. In all cases, immunohistochemical staining for the presence of p16 protein was performed as an adjunct to histopathological evaluation to confirm the diagnosis and accurately distinguish high-grade intraepithelial lesions (CIN2/3) from low-grade or non-dysplastic changes. The interpretation of p16 immunostaining followed standard diagnostic criteria used in our department. The cytology results were then compared with the histopathological results obtained during this visit. All patients underwent gynecological examination and colposcopic assessment. Cases with clinical or diagnostic suspicion of endometrial, ovarian, or tubal malignancy were excluded to ensure that abnormal cytology findings reflected lesions originating from the cervix or vagina.

### 2.2. Inclusion Criteria

- Age ≥ 50 years.
- Abnormal cytology result: HSIL, ASC-H or AGC diagnosed between 2018 and 2024, as the reason for referral to the Cervical Pathology Clinic at the Clinical Outpatient Clinic.
- Completion of in-depth diagnostics, including histopathological verification.

### 2.3. Statistical Analysis

A chi-square test of independence was performed to assess the relationship between selected clinical variables and histopathological results. A significance level of α = 0.05 was adopted.

The following hypotheses were formulated:

**H_0_** **(null** **hypothesis).** *the variables are independent, i.e., there is no relationship between clinical features and cytology accuracy.*

**H_1_** **(alternative** **hypothesis).** *the variables are dependent—there is a relationship between the clinical variable and the cytology result.*

Statistica 13.1 was used for data analysis.

## 3. Results

Table 1 presents the results of histopathological verification in women with abnormal cytology results (HSIL, ASC-H, and AGC). Comprehensive patient-level demographic and clinical characteristics, including pregnancy and menopausal status, smoking history, and immunosuppressive therapy, are provided in Supplementary Materials.

**Figure 1:**
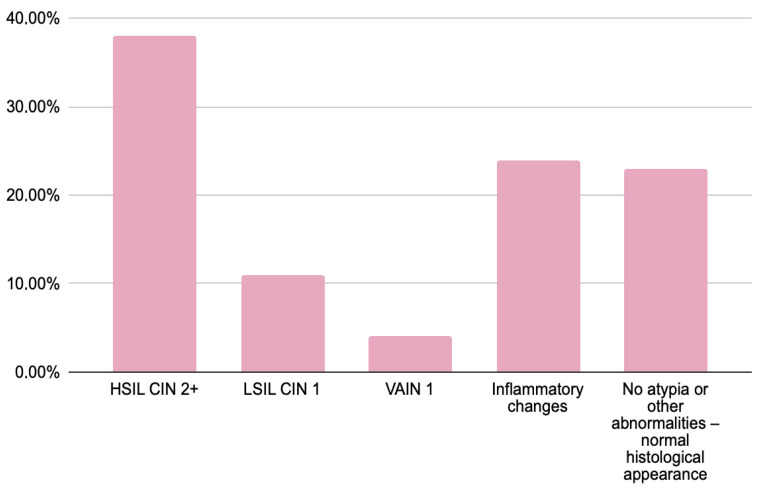
Results of histopathological verification in women with abnormal cytology findings (HSIL, ASC-H and AGC).

Figure 1 presents the distribution of histopathological outcomes in the study population.

The histopathological examination results of women with abnormal cytology results (HSIL—75%, ASC-H—23%, and AGC—2%) were analyzed. HSIL/CIN 2+ was confirmed in 30 women (38%), while VAIN was diagnosed in 3 (4%). In 9 women (11%), histology revealed less severe lesions than suggested by cytology, specifically LSIL/CIN 1. The majority of patients (37 women; 47%) showed either inflammatory changes or no significant pathology. Notably, HSIL/CIN 2+ was most frequently observed in women with a cytological diagnosis of HSIL, whereas those with AGC predominantly exhibited LSIL or no atypia upon histological examination.

**Figure 2:**
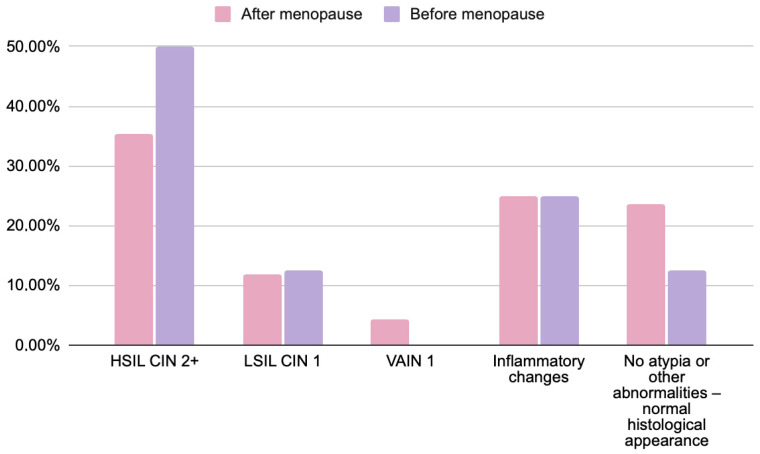
Comparison of histopathological findings in women before (*n* = 8) and after menopause (*n* = 68) with abnormal cytology results (HSIL, ASC-H, and AGC). Chi-square test; *p* = 0.2678. Data are presented as percentages. No statistically significant differences were found between groups. Note: menopausal status data were available for 76 of 79 participants due to incomplete medical records.

Figure 2 presents the comparison of histopathological outcomes between premenopausal and postmenopausal women.

Across menopausal subgroups, the distribution of histopathological outcomes showed minor numerical variations that were not statistically significant. HSIL/CIN2+ was observed in 35% of postmenopausal and 50% of premenopausal women, whereas LSIL/CIN1 occurred with similar frequency in both groups (12% vs. 12.5%). VAIN1 lesions were only diagnosed in postmenopausal patients (4.5%). Inflammatory changes and the absence of atypia occurred at comparable rates between groups. These findings confirm that menopausal status did not significantly affect the concordance between cytological and histological results (*p* > 0.05).

**Figure 3:**
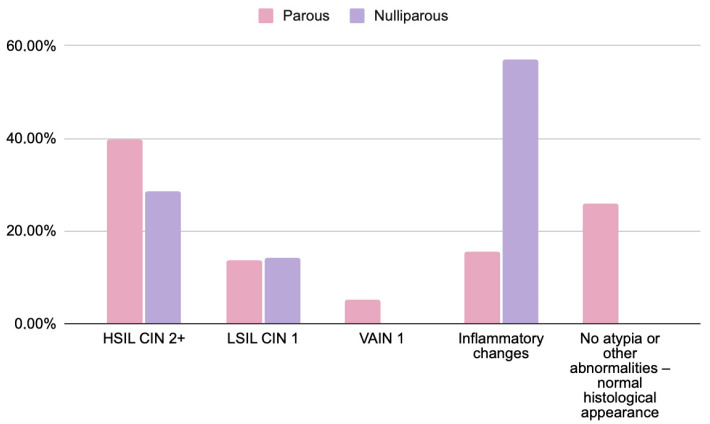
Comparison of histopathological findings in women based on obstetric history: parous (*n* = 58) and nulliparous (*n* = 7) patients with abnormal cytology results (HSIL, ASC-H, and AGC). Chi-square test; *p* = 0.4844. Data are presented as percentages. No statistically significant differences were found. Note: complete obstetric history data were available for 65 of 79 participants due to incomplete medical records.

Figure 3 presents the comparison of histopathological outcomes between parous and nulliparous women.

No statistically significant differences were found between parous and nulliparous women. While small numerical variations were noted (e.g., HSIL/CIN2+ observed in 39.5% vs. 29%), these results should be interpreted cautiously given the limited number of nulliparous participants. The frequency of inflammatory or low-grade lesions (LSIL, VAIN1) appeared comparable between groups.

The small number of smokers (*n* = 3) and immunosuppressed patients (*n* = 4) precluded reliable statistical analysis. Descriptive observation suggested a tendency toward more inflammatory changes among smokers and a higher proportion of HSIL among immunosuppressed women; however, these findings lack statistical significance (*p* > 0.05) and should not be considered clinically conclusive.

## 4. Discussion

This study revealed significant discrepancies between cytological and histopathological findings in patients with suspected HSIL. Only 38% of cytological diagnoses of HSIL/ASC-H were confirmed histopathologically as HSIL (CIN2+), while the remaining cases included low-grade lesions (LSIL—11%), inflammatory changes (24%), or no atypia (23%). These discrepancies may be due to factors hindering correct interpretation of the cytological image, such as chronic cervicitis or LSIL coexisting with atrophy or subatrophy due to HPV infection.

In postmenopausal women, hormonal changes strongly influence the cytological appearance of the cervix. The decline in estrogen levels causes epithelial thinning and atrophy, which results in smears dominated by small parabasal cells with relatively large nuclei and little cytoplasm. These age-related changes, often accompanied by inflammation or cellular degeneration, can resemble abnormal findings such as atypical squamous cells or low-grade squamous intraepithelial lesions (LSIL) [24,25]. Atrophic smears are also frequently low in cellularity, and the presence of inflammatory or degenerative background material can further obscure diagnostic details. As a result, distinguishing between benign atrophic changes and true dysplasia may be difficult. These pitfalls can lead to both false-positive and false-negative results [26,27]. In our study, such limitations may partly explain why cytology tended to overestimate lesion severity in postmenopausal women.

Koilocytosis and LSIL may be caused by LR HPV infection without the potential to develop HSIL and cervical cancer. These findings indicate that conventional cytology tends to overestimate the severity of epithelial abnormalities, as many cytological diagnoses suggesting high-grade lesions were not confirmed histopathologically. This observation highlights the limited specificity of cytology, particularly in postmenopausal women, where atrophic and inflammatory changes may mimic dysplastic features and lead to false-positive interpretations. These results highlight the need for clinicians to be educated in the interpretation of cytology results, and to familiarize themselves with current guidelines, particularly in the context of integrating clinical data with diagnostic results.

According to the current guidelines of the Polish Society of Gynecologists and Obstetricians (PTGiP), patients with a positive result for high-risk HPV, particularly HPV types 16 and 18, should undergo further diagnosis and treatment at a specialist outpatient clinic before safely returning to routine screening after several consecutive normal cytology results.

Particular attention should be paid to the assessment of pathological changes in the vaginal walls. Although vaginal cancer is the rarest cancer of the reproductive organs and the study group is not large, precancerous VAIN1 lesions were found in 4% of patients, which was an unexpected result resulting from a thorough evaluation of the vaginal walls during colposcopy. The annual incidence of vaginal cancer is estimated to be between 0.2 and 0.3 cases per 100,000 women [28]. It accounts for around 0.4% of all intraepithelial neoplasms of the lower reproductive tract [29].

These results highlight the importance of a detailed assessment of the female reproductive organs, particularly in postmenopausal women, as atrophic changes can complicate both cytological interpretation and colposcopic evaluation [30]. Colposcopy, and in particular the Schiller test, may play a key role in such cases.

In postmenopausal women, physiological atrophy of the cervix and declining estrogen levels also significantly reduce the diagnostic accuracy of colposcopy. The squamocolumnar junction (SCJ), which represents the most common site of high-grade lesions, often retracts into the endocervical canal, making complete visualization difficult [31]. In addition, atrophic changes in the cervical and vaginal epithelium lead to thinning of the squamous layer and decreased reactivity to acetic acid, while the reduced glycogen content results in weaker Lugol’s iodine uptake. These factors can cause lesions to appear atypical or indistinct from normal tissue, increasing the likelihood of missed or false-positive diagnoses. Furthermore, vaginal atrophy and reduced elasticity may hinder adequate exposure of the cervix during colposcopy, increasing procedural difficulty and patient discomfort [32].

To minimize the impact of postmenopausal atrophy on colposcopic assessment, several clinical strategies can be considered. One of the most effective and well-established approaches is short-term local estrogen therapy. Topical estrogen preparations restore epithelial thickness, increase vascularization, and replenish glycogen levels in the vaginal and cervical mucosa, thereby improving tissue elasticity and the visibility of the transformation zone [33]. These changes enhance the contrast between normal and abnormal epithelium under acetic acid and Lugol’s iodine staining, facilitating a more accurate diagnosis. Moreover, local estrogen administration helps normalize the vaginal microenvironment and reduce inflammation, both of which can improve the quality of colposcopic and cytological evaluation [34]. Therefore, the use of local estrogen therapy prior to colposcopy in postmenopausal women may represent a practical strategy to optimize diagnostic accuracy and reduce the likelihood of false-negative results.

Taking clinical factors into account, the analysis revealed no statistically significant differences in histopathological verification results. Although descriptive percentage differences were observed between subgroups, these were not confirmed by statistical tests (*p* > 0.05). This underlines the importance of interpreting the figures with caution and considering the limitations associated with the size of the groups being compared.

Among postmenopausal women, the incidence of HSIL was lower (35% compared to 50% in premenopausal women). Accordingly, benign findings without atypia were proportionally more common in postmenopausal women (23.5% vs. 12.5%). Despite the observed differences between the groups, they did not reach statistical significance (*p* = 0.2678), which limits the ability to interpret these results as being dependent on the analyzed feature. Confirmation of the potential trend would require studies on a larger sample size.

Although parous women had a higher proportion of HSIL lesions than nulliparous women (39.5% vs. 29%), this difference was not statistically significant (*p* = 0.4844), limiting the ability to draw definitive conclusions. The higher rate of inflammatory lesions observed in nulliparous women is likely incidental and should not be overinterpreted, as inflammatory changes are common and typically lack diagnostic or prognostic significance in the context of cervical neoplasia. The potential influence of parity on lesion characteristics warrants further investigation in larger cohorts.

Due to the small sample size of women who smoked (*n* = 3), it was not possible to draw reliable conclusions concerning the relationship between tobacco use and histopathological findings. Statistical analysis revealed no significant association between smoking status and cytology accuracy (*p* = 0.7148). Nevertheless, these data could form the basis for future research involving a larger sample size. Consequently, a more reliable analysis can be performed on the group of non-smoking patients, where the distribution of results was more stable. Despite the small size of the smoking group, the findings are consistent with previous reports on the role of smoking in increasing the risk of persistent HPV infection and HSIL development.

The incidence of HSIL was significantly higher in the group of immunosuppressed patients (75% vs. 36% in the non-immunosuppressed), though this difference did not reach statistical significance (*p* = 0.4456). However, the small number of patients in this subgroup limits the generalisability of the results. Nevertheless, this highlights the need to design and implement specialized diagnostic and follow-up pathways for immunocompromised women, involving more frequent surveillance than in the general population.

The main limitation of this study is the relatively small number of patients included in the analysis, particularly within clinical subgroups. This limited sample size reduces the statistical power and generalisability of the findings and may obscure potential associations between cytological accuracy and clinical characteristics such as menopausal status, parity, smoking, or immunosuppression. Consequently, all observed trends should be interpreted with caution.

This study was a pilot study conducted at a single centre which currently did not have access to a larger number of patients meeting the inclusion criteria. Therefore, expanding the research to a multicentre scale is recommended, as this would allow for an increased sample size and the generation of more representative results.

Despite the lack of statistical significance in the analysis of clinical characteristics (*p* > 0.05), a simulation was conducted using statistical tools to assess the impact of a tenfold increase in cases on outcome proportions. In this model, clinical features such as menopausal status, smoking status, pregnancy history, and immunosuppression were found to influence the accuracy of cytology. This suggests that with a sufficiently large sample size, it may be possible to detect significant associations that currently remain undetectable due to limited statistical power.

The findings support the critical role of histopathological verification of cytological abnormalities, particularly in cases suspected to be HSIL. At the same time, they emphasize the need for cautious interpretation of cytology results in a clinical context, and highlight the importance of considering factors that may affect diagnostic reliability. Diagnosis of cervical precancerous lesions should not rely on a single test, but rather require a holistic approach including histopathological verification and long-term patient monitoring, particularly for high-risk populations.

The high proportion of cases in which inflammatory changes (24%) or no atypia (23%) were diagnosed despite abnormal cytology results highlights the need to verify cytology results using more sensitive methods, such as HPV testing or molecular biomarkers. In postmenopausal women, inflammatory or atrophic changes may significantly obscure cytological interpretation, leading to both false-positive and false-negative results. Chronic cervicitis or LSIL coexisting with subatrophy can mimic high-grade lesions, posing a diagnostic challenge for cytotechnologists. In such cases, the use of Meigs’ test prior to cytological sampling could improve diagnostic reliability and should be considered in future screening strategies for this population.

The discrepancies between cytology and histopathology findings emphasize the importance of critically interpreting results and continually improving screening programmes, particularly for women aged ≥ 50 years.

Although the present study was limited to women with abnormal cytology results (HSIL, ASC-H, and AGC) and did not evaluate cervical cytology as a population-based screening method, it provides clinically relevant insight into the diagnostic utility of conventional cytology in women aged ≥50 years. Because the study population consisted exclusively of women referred for abnormal cytology, it does not allow for an assessment of cytology’s screening performance in the general population. The results instead reflect the positive predictive value of cytology among high-risk referrals.

The observed discrepancies between cytological and histopathological findings demonstrate that conventional cytology alone may have limited specificity in this age group, particularly in postmenopausal women with atrophic or inflammatory changes. From a clinical standpoint, these findings emphasize that in women aged ≥50 years, abnormal cytology findings should always be interpreted within the context of hormonal status, inflammatory background, and colposcopic visibility. Integration of molecular HPV testing and dual-stain (p16/Ki-67) cytology could represent a more reliable approach to triaging abnormal results in this population.

From a research perspective, future multicenter studies with integrated HPV genotyping, hormonal status assessment, and biomarker analysis are warranted to validate these findings and guide the development of evidence-based diagnostic algorithms for postmenopausal women.

In conclusion, this study underscores the importance of histopathological verification before invasive treatment and supports incorporating molecular methods to enhance diagnostic precision and improve patient management. The findings highlight the need to refine diagnostic algorithms and tailor cervical cancer screening strategies to the specific challenges of the postmenopausal population.

## Figures and Tables

**Table 1:** Correlation between cytological and histopathological findings in women aged ≥50 years with abnormal cytology results (HSIL, ASC-H, and AGC).

Cytology Results	HSIL/CIN2+	LSIL/CIN1	VAIN1	Inflammatory/No Atypia
HSIL (*n* = 59)	28 (47.5%)	5 (8.5%)	1 (2%)	25 (42%)
ASC-H (*n =* 18)	2 (11%)	3 (17%)	2 (11%)	11 (61%)
AGC (*n* = 2)	0 (0%)	1 (50%)	0 (0%)	1 (50%)
Total (*n* = 79)	30 (38%)	9 (11%)	3 (4%)	37 (47%)

HSIL—high-grade squamous intraepithelial lesion; LSIL—low-grade squamous intraepithelial lesion; ASC-H—atypical squamous cells, cannot exclude HSIL; AGC—atypical glandular cells; CIN—cervical intraepithelial neoplasia; VAIN—vaginal intraepithelial neoplasia.

## Data Availability

The datasets used and/or analyzed during the current study are available from the corresponding author upon reasonable request.

## Supplementary Materials

The following supporting information can be downloaded at: https://www.mdpi.com/article/10.3390/jcm14238305/s1.
