# Supplementary material for: Histopathological Verification of Abnormal Cytology Results Suggesting High-Grade Intraepithelial Lesions in Women over 50 Years of Age—Evaluation of the Clinical Utility of Conventional Gynecological Cytology
Source: J Clin Med. 2025 Nov 22;14(23):8305. doi: 10.3390/jcm14238305 (PMC12692454; doi:10.3390/jcm14238305)
Supplement: Supplementary file 1 [file jcm-14-08305-s001.zip › jcm-3963261-supplementary.pdf]

Table S1. summarizes the results of histopathological verification in women with abnormal cytology results (HSIL, ASC-H, and AGC).

PATIENT	CYTOLOGY RESULTS	HISTOPATHOLOGY RESULTS	MENOPAUSE	PAROUS/NULLIPAROUS	SMOKING	IMMUNOSUPPRESSION
Patient001	HSIL	No atypia	1	1	0	0
Patient002	HSIL	HSIL/CIN 2	1	0	0	0
Patient003	AGC	No atypia	1	1	0	0
Patient004	HSIL	HSIL/CIN 3	1	1	0	0
Patient005	HSIL	No atypia - inflammatory changes	1	0	0	0
Patient006	HSIL	HSIL/CIN 3	1	NA	0	0
Patient007	HSIL	No atypia - inflammatory changes	1	1	0	0
Patient008	HSIL	No atypia	1	1	0	0
Patient009	ASC-H	No atypia	0	1	0	0
Patient010	HSIL	LSIL	1	1	0	1
Patient011	HSIL	HSIL/CIN 3	0	1	0	0
Patient012	HSIL	HSIL/CIN 3	1	1	0	0
Patient013	HSIL	No atypia	1	1	1	0
Patient014	HSIL	HSIL/CIN 3	1	1	0	0
Patient015	AGC	LSIL	0	1	0	0
Patient016	HSIL	No atypia	1	1	0	0
Patient017	ASC-H	No atypia	1	1	0	0
Patient018	HSIL	No atypia	1	1	0	0
Patient019	HSIL	No atypia	1	1	0	0
Patient020	ASC-H	No atypia	1	1	0	0
Patient021	ASC-H	No atypia	1	1	0	0
Patient022	ASC-H	No atypia	1	1	0	0
Patient023	ASC-H	No atypia	1	NA	0	0
Patient024	ASC-H	HSIL	1	1	0	1
Patient025	HSIL	HSIL/CIN 2	NA	NA	0	0
Patient026	HSIL	HSIL/CIN 2	1	1	0	0
Patient027	HSIL	HSIL/CIN 2	1	1	0	0
Patient028	HSIL	HSIL/CIN 2	1	0	0	0
Patient029	HSIL	HSIL/CIN 2	1	1	0	1
Patient030	HSIL	HSIL/CIN 2	1	1	0	1
Patient031	HSIL	HSIL/CIN 2	1	NA	0	0
Patient032	HSIL	HSIL/CIN 2	1	1	0	0
Patient033	ASC-H	HSIL/CIN 3	1	NA	0	0

Patient034	HSIL	HSIL/CIN 3	1	1	0	0
Patient035	HSIL	HSIL/CIN 3	1	1	0	0
Patient036	HSIL	HSIL/CIN 3	0	1	0	0
Patient037	HSIL	HSIL/CIN 3	1	1	0	0
Patient038	HSIL	HSIL/CIN 3	0	1	0	0
Patient039	HSIL	HSIL/CIN 3	1	1	0	0
Patient040	HSIL	HSIL/CIN 3	0	1	0	0
Patient041	HSIL	HSIL/CIN 3	1	1	0	0
Patient042	HSIL	HSIL/CIN 3	1	1	1	0
Patient043	HSIL	HSIL/CIN 3	1	1	0	0
Patient044	HSIL	HSIL/CIN 3	1	1	0	0
Patient045	HSIL	HSIL/CIN 3	1	1	0	0
Patient046	HSIL	HSIL/CIN 3	NA	NA	0	0
Patient047	HSIL	HSIL/CIN 3	1	1	0	0
Patient048	HSIL	LSIL	1	1	0	0
Patient049	ASC-H	LSIL	1	1	0	0
Patient050	ASC-H	LSIL	1	1	0	0
Patient051	ASC-H	LSIL	1	1	0	0
Patient052	HSIL	LSIL	1	0	0	0
Patient053	HSIL	LSIL	1	1	0	0
Patient054	HSIL	LSIL	1	1	0	0
Patient055	ASC-H	No atypia	1	NA	0	0
Patient056	HSIL	No atypia	1	1	0	0
Patient057	HSIL	No atypia	1	1	0	0
Patient058	HSIL	No atypia	1	1	0	0
Patient059	HSIL	No atypia	NA	NA	0	0
Patient060	HSIL	VAIN I	1	1	0	0
Patient061	ASC-H	VAIN I	1	1	0	0
Patient062	ASC-H	VAIN I	1	1	0	0
Patient063	ASC-H	No atypia - inflammatory changes	1	NA	0	0
Patient064	HSIL	No atypia - inflammatory changes	1	0	0	0
Patient065	HSIL	No atypia - inflammatory changes	1	1	0	0
Patient066	HSIL	No atypia - inflammatory changes	1	1	0	0
Patient067	HSIL	No atypia - inflammatory changes	1	NA	0	0
Patient068	HSIL	No atypia - inflammatory changes	0	NA	0	0

Patient069	HSIL	No atypia - inflammatory changes	1	NA	0	0
Patient070	HSIL	No atypia - inflammatory changes	1	NA	0	0
Patient071	HSIL	No atypia - inflammatory changes	1	NA	0	0
Patient072	ASC-H	No atypia - inflammatory changes	1	1	0	0
Patient073	ASC-H	No atypia - inflammatory changes	1	0	0	0
Patient074	HSIL	No atypia - inflammatory changes	0	0	0	0
Patient075	ASC-H	No atypia - inflammatory changes	1	1	0	0
Patient076	HSIL	No atypia - inflammatory changes	1	1	0	0
Patient077	HSIL	No atypia - inflammatory changes	1	1	0	0
Patient078	HSIL	No atypia - inflammatory changes	1	1	0	0
Patient079	HSIL	No atypia - inflammatory changes	1	1	1	0

NA – data not available in patient records.
